# Possible Solution of the Fly Problem

**Published:** 1914-05

**Authors:** 


Possible Solution of the Fly Problem.—A trap to destroy the maggots of the typhoid or housefly before they develop into winged insests is a possible solution of the fly problem and one that should interest health officers, sanitarians, and others who might make use of it on manure heaps where this common pest breeds. The Department of Agriculture's scientists have succeeded in destroying from 70 to 95 per cent of the maggots in a pile of manure. A large galvanized iron pan, measuring five by three feet, with sides four inches high, was made. In this stood a container on legs eight inches high. This container measured four by two by two feet. The sides and bottom were of heavy wire, one-quarter-inch mesh, supported by a light wooden framework. Twelve cubic feet of manure well infested with eggs and larvæ were placed in this container and sprinkled with water. Water was also poured into the pan below to the depth of about one inch. Surrounding and covering both pan and container was a fly tight enclosure made of a large cage, six by six by six feet. This prevented further infestation of the manure, and an arrangement of traps at the top of the cage made it possible to capture and keep a record of any flies that might emerge. At the time for the emergence of flies the sides of the cage were darkened with black cloth in order to drive the flies into the traps at the top. Each day the maggots were collected from the pan and counted, and each day the manure in the container was sprinkled thoroughly with water and the pan was washed out and again partly filled with water to drown the larvæ which fell into it. From 98 to 99 per cent of the maggots were destroyed, if the manure was kept moist; from comparatively dry manure about 70 per cent were destroyed.—*New York Medical Journal.*

---

Dr. Bernard Fantus, of Chicago, said there was need for considerable improvement in prescription writing. This could be secured if it was realized that prescription writing could not be taught by lectures and demonstrations; that the students must be drilled in prescribing. He believed that the best results could be obtained if a course on pharmacy and prescription writing was given before the work in pharmacology was taken up. The students should be made familiar in this course with the various classes of pharmaceutical preparations and their prescription. When the student then entered on his course in pharmacology, he was ready to write prescriptions from the very beginning, and he ought to be required to write prescriptions for each of the important drugs as they were studied, paying special attention to methods of pleasant and efficient administration. When the student finally advanced to the study of therapeutics, he should be required to write prescriptions from the standpoint of the effect. If the clinical instructors would do their duty and require students to write prescriptions for the remedies needed by the patients treated in hospitals and in dispensaries, the students would leave the medical college well trained in prescribing.

